# Paracrine Signaling in Cell–Biomaterial Interactions in Scaffold Vascularization: A Mini Review

**DOI:** 10.3390/biomimetics11070492

**Published:** 2026-07-14

**Authors:** Anisa Cole, Naznin Sultana

**Affiliations:** Texas Undergraduate Medical Academy, School of Public and Allied Health, Prairie View A&M University, Prairie View, TX 77446, USA

**Keywords:** scaffold vascularization, paracrine signaling, tissue engineering, mesenchymal stem cells (MSCs), angiogenesis, biomaterial microenvironment

## Abstract

Vascularization remains a fundamental bottleneck in tissue engineering, as the absence of functional vascular networks limits oxygen and nutrient delivery, resulting in necrotic cores and poor host integration. While structural scaffold design and cell sourcing have advanced considerably, emerging evidence indicates that paracrine signaling, rather than direct cell contact or scaffold architecture alone, is the primary driver of angiogenesis and vasculogenesis within engineered constructs. Key cell types, including endothelial cells (ECs) and mesenchymal stem cells (MSCs), engage in bidirectional paracrine crosstalk through the secretion of vascular endothelial growth factor (VEGF), angiopoietins, hepatocyte growth factor, and platelet-derived growth factor, among other mediators. While researchers have long focused on improving scaffold structure and cell selection, growing evidence shows that the chemical messages cells send to one another play a far more important role in driving blood vessel formation than previously appreciated. This review explores how cells embedded within engineered scaffolds communicate through secreted signals to coordinate the growth of new blood vessels. Two cell types, MSCs and ECs, are central to this process: cells that line blood vessels and bone marrow-derived stem cells. These cells exchange a variety of chemical messages that instruct neighboring cells to multiply, move, and organize into vessel-like structures. Importantly, the material properties of the scaffold itself, including its stiffness, surface texture, and degradation over time, influence the signals cells produce and how those signals spread through the tissue. Strategies to amplify paracrine signaling include growth factor-loaded delivery systems, hypoxic and genetic preconditioning of MSCs, and perfusion bioreactor culture. In vitro and in vivo evidence consistently demonstrates that coculture systems leveraging paracrine interactions produce superior vascular outcomes compared to single-cell or acellular constructs. Despite this progress, challenges related to signaling complexity, reproducibility, and clinical translation persist. Integration of transcriptomic and proteomic profiling, computational modeling, and machine learning approaches offers a path toward rationally designed scaffolds that recapitulate the spatiotemporal dynamics of native vascular signaling and ultimately support functional tissue regeneration.

## 1. Introduction

The long-term success of tissue-engineered constructs depends on their ability to establish a functional vascular network [1,2]. Without adequate vascularization, engineered tissues suffer from insufficient oxygen and nutrient transport, leading to necrosis and poor integration with host tissue [1]. Although advances in scaffold fabrication and cell sourcing have significantly improved construct design, vascularization remains a central bottleneck, particularly for thick or metabolically demanding tissues [2]. As a result, understanding the biological mechanisms governing vascular formation in engineered environments has become a critical focus in tissue engineering research.

**Figure 1:**
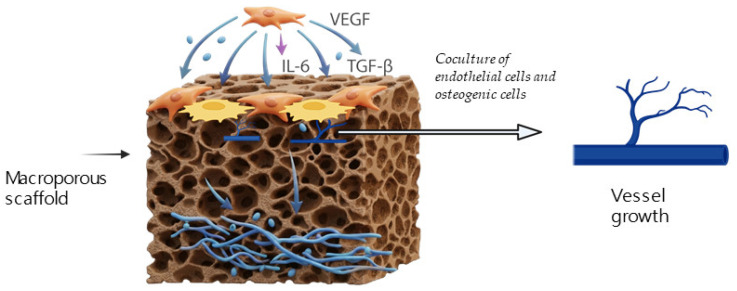
Method of Scaffold Vascularization via Paracrine Signaling. Cells seeded on a macroporous scaffold secrete the paracrine factors VEGF, IL-6, and TGF-β, which act on a coculture of endothelial and osteogenic cells to stimulate vessel formation, driving new vessel growth in the scaffold. Created in BioRender. Sultana, N. (2026) https://BioRender.com/v607mzi.

Early strategies to address this challenge largely emphasized structural solutions, such as increasing scaffold porosity, incorporating pre-formed channels, or seeding constructs with endothelial cells [3,4]. While these approaches improved perfusion in some contexts, they often failed to produce stable, mature vascular networks after implantation [5,6]. Increasing evidence now suggests that vascularization is not driven solely by cell presence or scaffold architecture, but by dynamic biochemical communication between cells and their surrounding materials [7]. In native tissues, angiogenesis and vasculogenesis are regulated through tightly coordinated signaling processes in which cells continuously exchange soluble cues that guide migration, proliferation, and differentiation [8]. Replicating these signaling environments within engineered scaffolds is therefore essential for achieving robust and durable vascularization [2]. Figure 1 illustrates the mechanisms by which scaffold vascularization is achieved through paracrine signaling, highlighting the key molecular interactions and cellular responses involved.

Paracrine signaling plays a central role in this process [8]. Cells embedded within or recruited to biomaterial scaffolds secrete growth factors, cytokines, and chemokines that act locally to influence endothelial behavior and vascular patterning [9]. These signals do not operate in isolation. Instead, their production, spatial distribution, and temporal dynamics are strongly influenced by the physicochemical properties of the surrounding biomaterial [10]. Scaffold stiffness, surface chemistry, degradation behavior, and microarchitecture can all modulate cellular phenotype and secretory profiles, thereby shaping the angiogenic microenvironment [7,10]. In this way, biomaterials function not only as structural supports but also as active regulators of intercellular communication [11].

Despite growing recognition of the importance of paracrine signaling in scaffold vascularization, the underlying mechanisms by which cell–biomaterial interactions regulate these signaling pathways remain incompletely understood [12]. Many studies report enhanced angiogenesis following specific material or coculture designs. However, fewer explicitly interrogate how biomaterial cues alter paracrine factor secretion, signaling pathway activation, or temporal coordination between cell populations [7,12]. This lack of mechanistic clarity limits the rational design of vascularized scaffolds and contributes to variability across experimental systems [2].

This review focuses on mechanistic insights into paracrine signaling in cell–biomaterial interactions that drive scaffold vascularization. We examine how key cell types, including endothelial cells and mesenchymal stem cells, communicate through soluble signaling pathways within engineered environments [9], and how biomaterial properties modulate these interactions [11]. By synthesizing current evidence across in vitro and in vivo studies, this review aims to clarify how paracrine signaling governs vascular network formation and to highlight design principles that can be leveraged to improve vascularization outcomes in tissue-engineered scaffolds.

## 2. Fundamentals of Scaffold Vascularization

**Figure 2:**
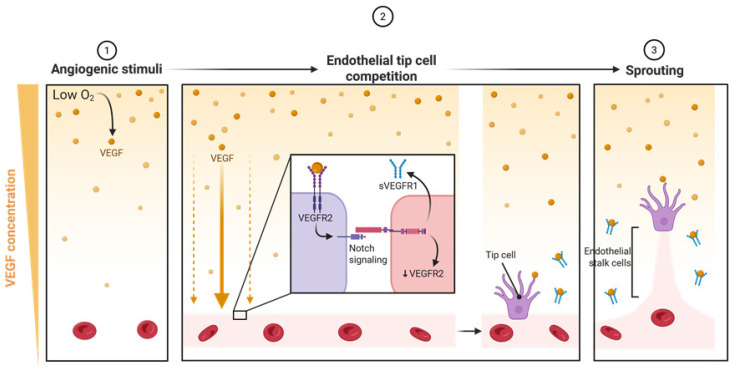
Angiogenesis process in a healthy blood vessel. (1) Angiogenic stimuli: Hypoxia (low O_2_) in surrounding tissue induces VEGF secretion, establishing a VEGF concentration gradient toward the existing blood vessel. (2) Endothelial tip cell competition: VEGF binds VEGFR2 on endothelial cells, activating Notch signaling, which upregulates soluble VEGFR1 (sVEGFR1) and downregulates VEGFR2 in neighboring cells—restricting tip cell fate to the cell with the highest VEGFR2 signaling. (3) Sprouting: The selected tip cell extends filopodia and migrates toward the VEGF gradient, guiding trailing endothelial stalk cells to form a new vascular sprout. Created in BioRender. Sultana, N. (2026) https://BioRender.com/0psnf6o.

The formation of a functional vascular network remains a central challenge in tissue engineering. In native tissues, vascularization occurs through two primary processes: vasculogenesis and angiogenesis. Vasculogenesis refers to the de novo formation of blood vessels from endothelial progenitor cells during embryonic development. In contrast, angiogenesis involves the sprouting, branching, or remodeling of existing vasculature and predominates in adult tissues (Figure 2). These processes are orchestrated by a complex interplay of molecular cues, among which vascular endothelial growth factor (VEGF) plays a pivotal role by regulating endothelial cell proliferation, migration, and survival [8,13]. Hypoxic tissue (low oxygen, O_2_) releases VEGF, forming a concentration gradient that increases away from the existing blood vessel. Endothelial cells lining the vessel sense this gradient via VEGFR2 (Vascular Endothelial Growth Factor Receptor 2); the cell with the highest VEGFR2 activation becomes the “tip cell,” which activates Notch signaling, specifically through its ligand DLL4 (Delta-Like Ligand 4), in its neighboring cells. This DLL4-Notch signaling suppresses VEGFR2 and upregulates sVEGFR1 (soluble Vascular Endothelial Growth Factor Receptor 1), a decoy receptor that sequesters VEGF, in adjacent cells, preventing them from becoming tip cells. The selected tip cell then extends filopodia and sprouts toward the VEGF source, while the neighboring “stalk cells” follow behind to elongate the new vessel branch.

**Figure 3:**
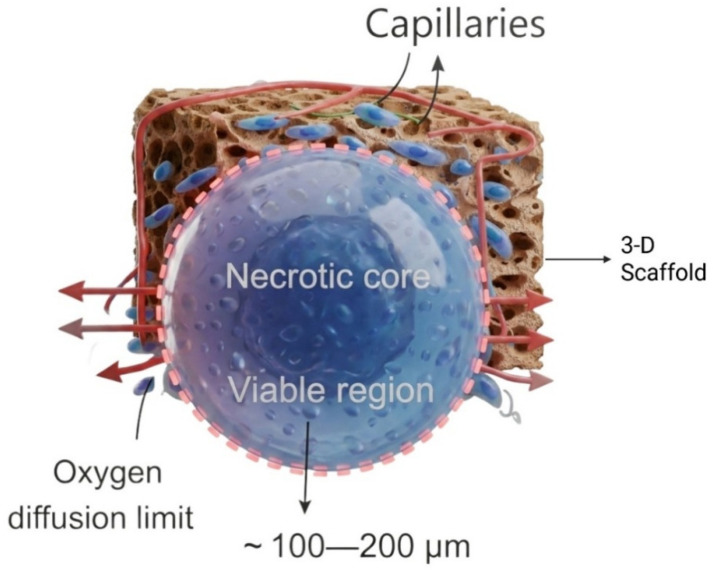
Diffusion Limitation in Scaffolds. Created in BioRender. In a 3-D scaffold with peripheral capillaries, oxygen/nutrient diffusion is limited to ~100–200 μm, maintaining a viable cell region near the surface while cells beyond this distance form a necrotic core due to insufficient supply. Created in BioRender. Sultana, N. (2026) https://www.biorender.com/gtr7wga.

In engineered constructs, vascularization remains a persistent barrier to the successful regeneration of thick or metabolically demanding tissues. Due to the limited diffusion range of oxygen and nutrients, cells within the core of avascular scaffolds are often necrotic approximately 100 to 200 µm from a capillary [4]. Figure 3 shows the diffusion limitations in scaffolds, which represent a critical barrier in tissue engineering, as the inability to transport oxygen and nutrients throughout the scaffold architecture efficiently leads to cell death and compromised tissue formation in regions beyond the diffusion threshold.

Moreover, even when angiogenic signals are incorporated into scaffolds, integration with the host vasculature via anastomosis can be delayed or incomplete, impairing long-term graft survival and function. Strategies such as prevascularization, coculture systems, and spatiotemporal delivery of growth factors have improved outcomes, but translation to clinical settings remains hindered by challenges related to immune responses, vascular stability, and control over neovascular patterning.

## 3. Paracrine Signaling in Cell–Biomaterial Interactions

A growing body of evidence suggests that the regenerative potential of cell-based therapies is largely mediated through paracrine signaling rather than direct cell integration. Paracrine signaling involves the local release of bioactive factors by cells that modulate the behavior of neighboring cells within the tissue microenvironment. In engineered tissues, this mode of communication governs key regenerative processes, including angiogenesis, immune modulation, and extracellular matrix remodeling [14].

Table 1 shows the key cell types and their paracrine contributions in scaffold vascularization. Multiple cell types contribute to paracrine signaling within scaffolds. Endothelial cells, for instance, are a primary source of VEGF and angiopoietins that initiate vascular sprouting and lumen formation [15]. Mesenchymal stem cells (MSCs), widely used in regenerative medicine, exert broad paracrine effects by secreting cytokines, chemokines, and growth factors that influence not only endothelial behavior but also fibroblast recruitment, macrophage polarization, and matrix deposition [16]. In bone tissue engineering contexts, osteoblasts contribute to both osteogenesis and angiogenesis by releasing interleukin-6 (IL-6), bone morphogenetic proteins, and other osteoinductive factors [17].

**Figure 4:**
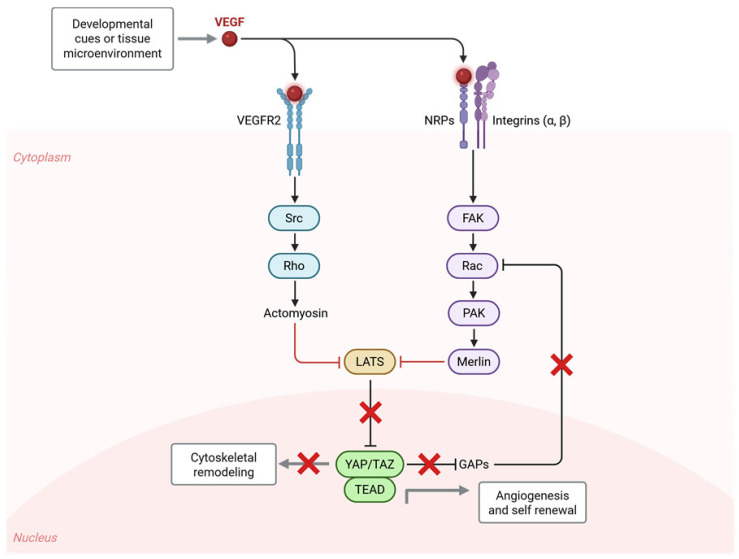
Angiogenesis process within the cells. VEGF activates two parallel signaling branches: VEGFR2 triggers Src-Rho-mediated actomyosin activity, while NRP/integrin complexes trigger FAK-Rac-PAK-Merlin signaling. Both branches converge to inhibit LATS, blocking the Hippo pathway’s suppression of YAP/TAZ. This allows YAP/TAZ-TEAD to remain active in the nucleus, driving cytoskeletal remodeling, GAP expression, and transcription of genes promoting angiogenesis and cell self-renewal. Created in BioRender. Sultana, N. (2026). https://BioRender.com/0psnf6o.

The biophysical and biochemical properties of scaffolds themselves can modulate paracrine signaling. Surface chemistry governs protein adsorption and cell adhesion, thereby altering the secretory profile of attached cells [18]. Figure 4 shows the angiogenesis process within the cells. VEGF activates two receptor systems, VEGFR2 and a co-receptor complex of NRPs (Neuropilins) with Integrins (α and β subunits), which signal through parallel cascades: VEGFR2 via Src (Src proto-oncogene tyrosine kinase) → Rho (Ras Homolog family member GTPase) → actomyosin, and NRPs/Integrins via FAK (Focal Adhesion Kinase) → Rac (Ras-related C3 botulinum toxin substrate GTPase) → PAK (p21-Activated Kinase) → Merlin (Moesin-Ezrin-Radixin-Like protein/Neurofibromin 2), both of which normally inhibit LATS (Large Tumor Suppressor kinase) and thereby restrain YAP/TAZ (Yes-Associated Protein/Transcriptional co-Activator with PDZ-binding motif); however, the red “X” marks indicate that these inhibitory checkpoints, including the GAPs (GTPase-Activating Proteins) feedback loops, are disrupted, allowing YAP/TAZ to escape suppression, partner with TEAD (TEA Domain transcription factor) in the nucleus, and drive gene expression that promotes angiogenesis and self-renewal. Substrate stiffness has been shown to influence mechanotransduction pathways, such as YAP/TAZ, which, in turn, regulate the expression of angiogenic cytokines, such as VEGF, and matrix metalloproteinases [19]. Additionally, nanoscale topographical features have been demonstrated to enhance the secretion of proangiogenic factors, indicating that scaffold microarchitecture can be harnessed to direct paracrine-mediated regeneration [20].

## 4. Major Paracrine Factors and Pathways

The paracrine factors involved in scaffold vascularization encompass a broad range of growth factors, cytokines, and chemokines, each acting through distinct but interconnected pathways. VEGF remains the most extensively studied, exerting potent effects on endothelial proliferation, permeability, and tube formation [13]. Fibroblast growth factor-2 (FGF-2) acts synergistically with VEGF to enhance endothelial proliferation and stabilize newly formed vasculature [21]. Platelet-derived growth factor (PDGF) contributes to vessel maturation by recruiting pericytes and smooth muscle cells to nascent capillaries [22], while transforming growth factor-beta (TGF-β) plays a dual role by promoting extracellular matrix deposition and modulating immune responses [23].

Proinflammatory cytokines such as IL-6 also exhibit proangiogenic activity, primarily through activation of the STAT3 pathway, which promotes endothelial cell survival and migration [24]. Chemokines such as monocyte chemoattractant protein-1 (MCP-1) recruit monocytes and macrophages to the injury site, where these immune cells subsequently release additional angiogenic factors and facilitate vessel remodeling [25]. These signaling molecules are tightly regulated and context-dependent, underscoring the importance of scaffold design in orchestrating their spatial and temporal presentation.

Underlying these paracrine responses are several well-characterized intracellular signaling cascades. Table 2 presents paracrine factors and signaling pathways along with their respective roles in vascularization. The PI3K/Akt pathway mediates survival and proliferation responses in endothelial cells, promoting nitric oxide production and vascular remodeling [26]. The MAPK/ERK pathway is activated by FGF and VEGF, regulating mitogenic and morphogenic outcomes [27]. Notch signaling, particularly via Delta-like ligand 4 (Dll4), governs endothelial cell fate decisions and branching morphogenesis during angiogenesis [28]. Furthermore, hypoxia-inducible factor 1-alpha (HIF-1α) is stabilized under low-oxygen conditions common in early scaffold environments and drives transcription of VEGF and other proangiogenic genes [29].

Together, these pathways represent a dynamic network of feedback and feedforward loops that determine the regenerative success of tissue-engineered constructs. Modulating these pathways through both biological and materials engineering approaches holds promise for advancing scaffold vascularization and ultimately improving clinical outcomes.

## 5. Mechanistic Insights

**Figure 5:**
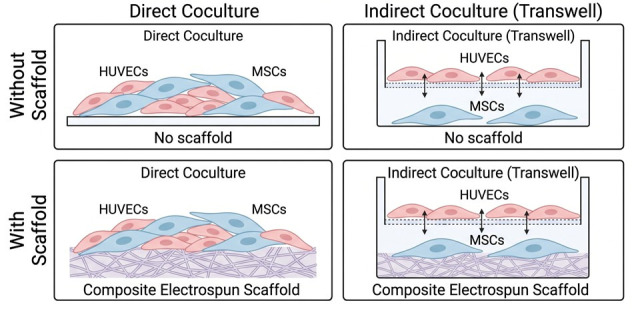
Strategy of four experimental groups and culture conditions for HUVECs and MSCs on composite electrospun scaffolds. Four culture configurations: (1) HUVEC + MSC direct coculture, (2) HUVEC + MSC indirect coculture (transwell, no scaffold), (3) HUVEC + MSC direct coculture on scaffold, and (4) HUVEC + MSC indirect coculture on scaffold (transwell), comparing direct versus paracrine-mediated HUVEC-MSC interactions with and without the scaffold. Created in BioRender. Sultana, N. (2026) https://BioRender.com/0nk8nyi.

Understanding the mechanisms by which paracrine signaling mediates scaffold vascularization requires examining the cellular crosstalk between key cell types, the regulatory influence of biomaterials, and the temporal progression of signaling events. Among these, the interactions between mesenchymal stem cells (MSCs) and endothelial cells (ECs) are especially critical. MSCs have been shown to enhance endothelial tube formation by secreting VEGF, hepatocyte growth factor (HGF), and angiopoietin-1, even without direct contact. Conversely, ECs can influence MSC differentiation and survival by releasing nitric oxide and extracellular vesicles containing regulatory microRNAs [30,31]. This bidirectional paracrine crosstalk underpins the cooperative behavior observed in many coculture systems (Figure 5). This figure compares four HUVEC (Human Umbilical Vein Endothelial Cell)-MSCs coculture configurations: in “Direct Coculture,” both cell types are seeded together as a mixed monolayer in direct physical contact at the bottom of a well; in “Indirect Coculture,” a transwell insert separates an MSC layer on the porous membrane from a HUVEC layer at the well bottom, allowing only soluble-factor (paracrine) communication without direct contact; in “Direct Coculture on Scaffold,” no insert is used, and a porous scaffold is seeded with HUVECs on its lower surface and MSCs on its upper surface, placing both cell types in direct contact through the scaffold; and in “Indirect Coculture on Scaffold,” a transwell insert holds the MSC layer above a scaffold-covered HUVEC layer at the well bottom, enabling diffusion-based crosstalk across the scaffold while keeping the two cell populations physically separated.

Scaffold properties strongly modulate these interactions. Material stiffness can regulate cell phenotype and secretory activity through mechanotransduction pathways such as integrin-FAK signaling and YAP/TAZ nuclear localization [19,32]. Porosity and degradation rate affect nutrient diffusion and the dynamic release of embedded factors, while biochemical functionalization of scaffolds can sequester or present paracrine cues in a spatially organized manner [7]. Thus, the material microenvironment acts not merely as a passive support, but as an active regulator of cell signaling.

The temporal dynamics of paracrine signaling also play a vital role in neovascularization. Early stages are characterized by proangiogenic factors, such as VEGF, IL-8, and MCP-1, which promote endothelial migration and sprouting. As vascular structures stabilize, the secretion of anti-permeability and maturation cues such as angiopoietin-1, PDGF-BB, and TGF-β increases [33]. Mimicking this time-dependent pattern through biomaterial and cell-based engineering is a key goal in achieving functional vascular networks.

## 6. Strategies to Enhance Paracrine Signaling

To harness paracrine signaling effectively, both material and biological strategies have been developed. One widely adopted approach is to incorporate bioactive molecules directly into scaffolds via immobilization or encapsulation, allowing for localized and sustained release. For instance, VEGF-loaded hydrogels or microspheres can recreate concentration gradients that mimic physiological angiogenesis [34]. Moreover, scaffolds functionalized with peptides such as RGD or heparin can promote cell adhesion and retain growth factors within the matrix [35].

On the cellular side, preconditioning strategies have shown promise in enhancing paracrine potency. Hypoxic preconditioning of MSCs, for example, leads to upregulation of HIF-1α and subsequent increases in VEGF, IL-6, and angiogenin secretion [36]. Genetic modification offers another avenue, with MSCs engineered to overexpress proangiogenic genes such as VEGF or Ang-1, resulting in superior vascular outcomes [37]. While these approaches enhance secretion, they must be carefully evaluated for safety, immunogenicity, and regulatory compliance.

Dynamic culture systems further augment paracrine signaling by providing mechanical and biochemical cues. Perfusion bioreactors improve oxygen and nutrient transport while introducing shear stress, which enhances endothelial differentiation and VEGF secretion [38]. Mechanical stimulation, such as cyclic strain or compression, has also been shown to upregulate the release of angiogenic factors from both MSCs and osteoblasts, suggesting that biophysical stimuli can be leveraged as therapeutic inputs [39].

The pathways, scaffold properties, and strategies discussed above target vascularization at different levels and timescales. Various signaling cascades control a hypoxia-driven master switch, the early sprouting phase, and Notch/DLL4 acts later to restrain tip-cell selection and coordinate branching, meaning interventions targeting these pathways address sequential rather than competing stages. Scaffold properties show a similar division: porosity and degradation rate govern fast, diffusion-based factor delivery, stiffness modulates slower YAP/TAZ-driven phenotypic changes, and surface functionalization (e.g., RGD, heparin) bridges the two by controlling adhesion and localized factor retention. Enhancement strategies likewise trade off differently; material-based approaches (encapsulation, gradients) offer strong spatial control but limited post-implantation flexibility, cell-based approaches (preconditioning, genetic modification) offer strong secretory control but less spatial precision, and dynamic culture systems (bioreactors, mechanical stimulation) balance both at the cost of added complexity. Overall, no single pathway, scaffold parameter, or strategy is sufficient alone; robust vascularization likely requires combinatorial designs pairing early HIF-1α-driven cues with spatially controlled scaffold gradients and late-stage maturation signals like PDGF and TGF-β.

## 7. Current Advances and Experimental Evidence

A growing body of experimental evidence has substantiated the central role of paracrine signaling in scaffold vascularization. In vitro coculture systems have shown that mesenchymal stromal cells (MSCs) and endothelial cells (ECs) seeded together on decellularized or perfusion-cultured scaffolds achieve more robust, stable endothelialization than ECs cultured alone. This enhancement is largely attributable to MSC-derived paracrine mediators, particularly VEGF-A, which attenuate inflammatory activation and reduce endothelial cell death induced by perfusion-associated shear stress [40]. Notably, these beneficial effects are sustained even under conditions of limited direct cell–cell contact, supporting the conclusion that soluble signaling factors, rather than physical intercellular interactions, represent the principal drivers of the vascularization response.

Complementary in vivo evidence further underscores the functional significance of this paracrine crosstalk. Prevascularized constructs co-seeded with MSCs and ECs demonstrate superior perfusion and host integration relative to acellular scaffolds or those seeded with a single cell type. In immunodeficient murine models of hindlimb ischemia, implantation of pre-cultured, cell-encapsulating microbead constructs has been shown to accelerate inosculation with host vasculature, enhance macroscopic tissue reperfusion, and improve limb salvage outcomes compared with cellular controls lacking an established microvascular network prior to implantation [41].

Collectively, these findings support the primacy of secreted signaling molecules in driving angiogenesis within tissue-engineered constructs. Although direct intercellular contact mechanisms, such as gap junction communication, may exert modulatory effects, secreted paracrine factors remain the dominant determinant of vascular outcomes. This body of evidence lends strong support to the therapeutic rationale for cell-free strategies that recapitulate these paracrine mechanisms, including the use of bioprinted extracellular vesicles engineered to enable spatially controlled, localized delivery of regenerative cargo [42].

## 8. Challenges and Future Directions

Despite these advances, several challenges remain. The complexity of paracrine signaling networks, which involve redundant, overlapping, and sometimes antagonistic factors, complicates precise manipulation. Moreover, paracrine activity is highly context-dependent, influenced by cell source, culture conditions, and scaffold composition, making reproducibility across laboratories difficult. Standardizing protocols for cell isolation, scaffold fabrication, and in vivo assessment will be essential to improve comparability and reproducibility. Table 3 outlines the key challenges in tissue engineering and the corresponding strategies developed to address them.

Emerging technologies offer new opportunities to address these limitations. High-throughput “omics” approaches, including transcriptomics, proteomics, and secretomics, enable comprehensive profiling of paracrine factor networks in different scaffold environments [43]. Computational modeling and systems biology frameworks can integrate these datasets to predict optimal scaffold and culture conditions for vascularization. More recently, artificial intelligence and machine learning are being applied to design scaffolds with predictive control over cell behavior and paracrine dynamics, accelerating the design–build–test cycle in tissue engineering [44,45].

Despite mechanistic progress, several limitations constrain the reliability and clinical readiness of paracrine-based vascularization strategies. Reproducibility remains a persistent issue, as paracrine signaling networks involve redundant and sometimes antagonistic factors whose net effect is difficult to isolate or predict across different experimental systems. This is compounded by variability in cell sources, since MSCs and other paracrine-active cell populations differ in secretory profile depending on donor age, tissue origin, isolation method, and passage number, making outcomes inconsistent even within similar scaffold designs. Vascular stability presents an additional challenge, as newly formed vessels are often immature and prone to regression once growth factor delivery ceases, indicating that current strategies are better at initiating angiogenesis than sustaining it long-term. Finally, clinical translation is hindered by practical and regulatory barriers, including the complexity of manufacturing and quality control of cell-based or genetically modified constructs, uncertainty around the regulatory pathway for combination cell–biomaterial interactions, and the difficulty of scaling prevascularization or bioreactor-based approaches to clinically relevant sizes. Together, these limitations suggest that advancing beyond proof-of-concept studies will require not only mechanistic refinement but also standardized protocols for cell sourcing and scaffold fabrication, as well as strategies to promote long-term vascular maturation.

## 9. Conclusions

Vascularization remains a critical challenge in tissue engineering, and this review underscores that paracrine signaling, not scaffold architecture or direct cell contact alone, is the primary driver of angiogenesis in engineered constructs. Endothelial cells, MSCs, osteoblasts, and macrophages coordinate vessel formation through overlapping secretory factors and shared pathways, while scaffold properties shape these signaling dynamics rather than serving as passive supports. Strategies including growth factor delivery, MSC preconditioning, coculture systems, and bioreactor culture have improved vascular outcomes, yet reproducibility, immune compatibility, and scalability remain key translational hurdles. Advancing the field will require integrating omics profiling with computational and machine learning approaches to enable rationally designed scaffolds that better replicate native vascular signaling and support functional, clinically translatable vascularization.

## Figures and Tables

**Table 1:** Key cell types and their paracrine contributions in scaffold vascularization. Summary of the major cell types involved in scaffold vascularization, their key secreted paracrine factors, and their primary roles in driving angiogenesis and vascular integration within tissue-engineered constructs.

Cell Type	Key Secreted Factors	Primary Function in Vascularization
Endothelial Cells	Vascular endothelial growth factor (VEGF), Angiopoietins	Initiate vessel sprouting and lumen formation [8,13].
Mesenchymal Stem Cells (MSCs)	VEGF, releasing interleukin-6 (IL-6); Transforming growth factor β (TGF-β)	Support angiogenesis, modulate immune response, enhance tissue repair [9].
Osteoblasts	IL-6; Bone Morphogenetic Proteins (BMPs)	Promote osteogenesis and vascular coupling in bone tissue [17].
Macrophages	VEGF; Tumor Necrosis Factor Alpha (TNF-α), IL-1β	Regulate inflammation and angiogenic signaling [16].

**Table 2:** Paracrine factors, signaling pathways, and their role in vascularization. Summary of key paracrine factors involved in scaffold vascularization, their associated intracellular signaling pathways, and their specific roles in regulating endothelial cell behavior, vessel maturation, and vascular remodeling.

Paracrine Factor	Signaling Pathway	Role in Vascularization
Vascular endothelial growth factor (VEGF)	Phosphoinositide 3-Kinase (PI3K)/Protein Kinase B (Akt), Mitogen-Activated Protein Kinase (MAPK)/Extracellular signal-Regulated Kinase (ERK)	Promotes endothelial proliferation, migration, and tube formation [13,26].
Fibroblast growth factor-2 (FGF-2)	MAPK/ERK	Enhances angiogenesis and stabilizes vascular structures [21,27].
Platelet-derived growth factor (PDGF)	Platelet-Derived Growth Factor Receptor (PDGFR) signaling	Recruits pericytes and smooth muscle cells for vessel maturation [22,28].
transforming growth factor-beta (TGF-β)	Suppressor of Mothers against Decapentaplegic (SMAD) pathway	Regulates extracellular matrix deposition and vascular remodeling [23,29].
Interleukin-6 (IL-6)	Signal Transducer and Activator of Transcription 3 (STAT3) pathway	Supports endothelial survival and proangiogenic responses [17,29].
Monocyte chemoattractant protein-1 (MCP-1)	C-C Chemokine Receptor type 2 (CCR2) signaling	Recruits monocytes/macrophages to support angiogenesis [25].

**Table 3:** Challenges and engineering strategies to mitigate the challenges. Summary of key barriers to achieving functional vascularization in tissue-engineered scaffolds, along with corresponding engineering strategies designed to address each challenge and improve vascular network formation, stability, and integration with host tissue.

Challenge	Description	Engineering Strategy
Diffusion limitation	Oxygen/nutrient transport limited to ~100–200 µm	Prevascularization, microchannel design
Poor host integration	Delayed or incomplete anastomosis	Coculture systems, angiogenic factor delivery
Lack of vascular stability	Immature vessels regress over time	Controlled release of growth factors (e.g., VEGF, PDGF)
Immune response	Inflammation disrupts vascular formation	Immunomodulatory biomaterials
Spatial control of signaling	Difficulty replicating natural gradients	Gradient delivery systems, bioreactors

## Data Availability

No new data were created.
